# To debate or not to debate? Examining the contribution of debating when studying medical ethics in small groups

**DOI:** 10.1186/s12909-022-03124-0

**Published:** 2022-02-20

**Authors:** Nehora Amar-Gavrilman, Miriam Ethel Bentwich

**Affiliations:** 1 Tel Hai College, P.O.Box 1220800, Upper Galilee, Israel; 2 Faculty of Medicine, Bar-Ilan University, P.O. Box 1589, Safed, Israel

**Keywords:** Medical ethics learning, Educational debates, Small-group learning, Debating

## Abstract

**Background:**

Medical ethics is a significant learning topic for medical students, and often studied through small group learning (SGL) to encourage critical thinking (CT) and tolerance for ambiguity, both considered particularly important when coping with medical ethics dilemmas. However, a previous study about CT and tolerance for ambiguity in medical ethics SGL produced mixed results. Debating is a pedagogical tool known to enhance CT but never used before in medical ethics learning. This paper examines whether the use of debate may enhance medical ethics SGL by contributing to the CT of students and their tolerance of ambiguity.

**Methods:**

Intervention study using the qualitative microanalysis research method, based on videotaped observations that were analyzed through Kamin’s model of CT and non-CT. The study was conducted at Bar-Ilan University’s Faculty of Medicine in the years 2017–2019. Forty-four students and 4 facilitators participated, equally split between 4 small groups. Twenty-four medical ethics SGL sessions at the beginning and end of the year were videotaped, 2 groups – with no intervention, 1 group included partial debate intervention and 1 group fully used debates. Results were compared for changes in CT and ambiguity before and during the intervention period.

**Results:**

The full intervention (debating) group had the highest increase in utterances reflecting CT, thus actually doubling the median number of CT utterances at the end of the year in comparison to the median number at the beginning of the year. In a similar manner, the debate group exhibited the only group in which there was an increase in the median utterances of tolerance to ambiguity. Nevertheless, the debate group also exhibited the largest increase in the median non-CT utterances and the lowest decrease of intolerance to ambiguity, when comparing the end of the year to the beginning of the year sessions.

**Conclusions:**

Debating is an important enhancement to SGL in medical ethics; however, it does not guarantee a complete absence of non-CT.

## Introduction

Ethical conduct is a fundamental keystone of contemporary medical professionalism and is an integral and mandatory part of the curriculum in most medical schools [1–3]. Acknowledging the challenges entailed in addressing ethical issues, in the last 20–30 years, medical ethics has been taught with an emphasis on critical thinking skills, tolerance for ambiguity, and openness to differing viewpoints [4, 5].

Encouraging and enhancing critical thinking (CT) through small-group learning (SGL) is especially important when training medical students in medical ethics. According to a pivotal contemporary definition CT is “the intellectually disciplined process of actively and skillfully conceptualizing, applying, analyzing, synthesizing and/or evaluating information gathered from, or generated by, observation, experience, reflection, reasoning or communication, as a guide to belief and action.” ([6], p. 90) Understanding ethical dilemmas and their related complexities clearly necessitates the ability to examine them thoroughly in a reflective and open-minded manner, while also making decisions regarding these dilemmas. Doing so ultimately entails action guided by a precise and critical examination of them.

Applying CT skills also encourages tolerance for ambiguity. Thus, broadly speaking, tolerance for ambiguity revolves around acknowledging the existence of multiple interpretations for the same situation. Applying CT, among else, is precisely based on creating an atmosphere that allows for different opinions, thereby also setting the foundations for tolerance of ambiguity [7, 8].

Still, most of the studies conducted thus far to assess SGL use in learning medical ethics are less focused on any direct examination of the CT development process supposedly taking place in this particular type of learning. Instead, the previous studies were mainly based on students’ self-evaluation questionnaires or interviews with lecturers, most of which were not even focused on CT, but rather on the topic of medical ethics [9, 10].

Indeed, in a previous and unique study, designed precisely to examine the extent to which CT actually takes place in SGL sessions of medical ethics, mixed results were observed [11]. While 2/3 of the examined videotaped SGL showed clear utterances of CT by the medical students participating in the study, 1/3 of their utterances reflected non-CT statements. In other words, simply learning medical ethics through SGL might not be sufficient enough to encourage students to employ CT in their own deliberation of actual medical ethics dilemmas.

Alternatively, debating has been used in different fields of education and is a method that, among other aspects, to assist and encourage the use of CT. While the concept of debate in the West dates back to the ancient Greeks, in its current modern form, debate has become a formal framework where two sides discuss a topic using different perspectives [12]. Dozens of studies have been conducted over the last decade on integrating debates into various disciplines [13–19]. These studies, supposedly, presented a long list of skills that debate promote, including: open-mindedness, speed of response, communication strategies, public speaking, argumentative ability, the distinction between opinion and fact, listening, meaningful and experiential learning, group work and collaboration, self-confidence, improving the spoken language, clarifying values, ​​ formulating ideology and CT. Still, in these studies, the participants answered knowledge and satisfaction questionnaires without any reference being made to the students’ thinking processes. Further, no research has been conducted thus far on how medical students can integrate debating into their study and practice of medical ethics.

Against this backdrop, our study aims to evaluate the possible contributions of using debating to encourage and develop critical thinking (CT) in the context of delivering medical ethics small group learning (SGL).

## Method

### Research design

The current paper reports on the qualitative part of a larger mixed-method study, which aimed at examining debating in the context of SGL sessions on medical ethics, taking place at the Faculty of Medicine of Bar-Ilan University (Israel) in 2017–2019. The qualitative part is based on 24 videotaped observations of SGL sessions that covered 4 separate small groups, 2 of which underwent educational intervention (debating) as part of the videotaped observations. Additional details on the manner in which these observations and interventions were conducted are provided in the *Procedure* section below. Data analysis was conducted using the microanalysis approach, further detailed here in the *Data Analysis* section*.*

### Participants and sampling

In the qualitative portion of the study reported herein, 44 first-year students in our graduate program and 4 facilitators participated and were equally split into 4 groups for the SGL sessions on medical ethics. We utilized a convenience sampling since all the groups were drawn from the mandatory course in medical ethics and medical humanities taught by the second author of the paper. However, it should be emphasized that the coordination, recruitment, observations. and interventions done got the study, were conducted solely by the first author who was at the time a PhD student, not responsible for any of the students’ grades.

### Procedure

The study took place during the “Medical Humanities and Medical Ethics” longitudinal pre-clinical mandatory course for the Graduate (4-year) program at our university. For this study, we focused on first-year students, and specifically, 4 groups (out of 7) were videotaped while conducting SGL sessions in medical ethics. As a baseline, these sessions employ Case-Based-Learning (CBL), whereby the teaching materials (in medical ethics) are learned and applied through discussing particular (fictional) case that is relevant to the topic of study (e.g., cultural competence, ethics in reproduction and genetics, etc.). 2 (of the 4 videotaped groups) did not employ any intervention, so that they simply used the baseline CBL (please see a brief description in the next paragraph). One group with partial intervention was using the same baseline CBL method of learning but did have at the beginning of the year a single introductory class to debating and the manner in which it might assist the students during the rest of the CBL sessions. The remaining group, constituting the full intervention group,^1^ fully employed debating as part of its SGL sessions (as further described in the next paragraph). Overall, 6 sessions were videotaped; 2 at the beginning of the year, before the intervention and 4 at the end of the year, when debating was applied to the full intervention group and after the partial and full intervention groups had already received their introductory one-time session on debating.

In the SGL sessions that utilized CBL, the facilitator, together with the students, examined case studies that raised medical ethical dilemmas and analyzed them from various perspectives through guiding questions. In the full intervention (debating) group, the same cases as were reviewed in other groups’ sessions were used, but this group utilized a different procedure. The classroom was split into two areas, each signifying a general stance – either “for” or “against.” Students were then asked to assign themselves to either of these stances and sit accordingly in the designated area for such stance, without knowing yet the content of the case that they will be discussing. Then, the case that will be discussed was introduced and the students were asked to enumerate the considerations for the justification of the side that they represented “for” or “against”, regardless of their personal position and in accordance with the principles of medical ethics (autonomy, beneficence, non-maleficence, justice). Afterwards, the discussion of the case further proceeded, but without the assigned role that the students played previously, so that they could state their own opinion and the reasoning behind it.

The cases throughout the year dealt with ethical issues that were arising from a range of topics, such as cultural competence, allocation of public health goods, eugenics and genetic information law, termination of pregnancy and prenatal diagnosis, and ethical issues related to late-onset genetic syndromes and to designer babies.

### Data analysis

All 24 videotaped sessions were transcribed *ad verbatim* by the first author. The data analysis used a microanalysis approach, informed by a model for critical thinking that was suggested by Carol Kamin and her colleagues [20]. This model was specifically designed for clinical medical education and inspired and based on two previously developed separate pivotal models by Garrison and Newman on critical thinking [21, 22]. Kamin and her colleagues suggested five stages consisting of critical/CT (in their terms, “deep-level”) and non-critical/non-CT (in their terms, “surface-level”) thinking. These five stages are: (1) problem identification; (2) problem description; (3) problem exploration; (4) application; and (5) integration/critical assessment. Particular details regarding the various components of critical and non-critical thinking can be found in the article by Kamin and her colleagues [20]. In addition, we demonstrate how some of these components were applied using a short excerpt from our analysis in the “Results” section that follows.

Microanalysis is used in qualitative research in studies that are based and focused on detailed accounting for videotaped conversational episodes [23–26]. In such detailed account, the number of utterances of particular themes of interest per observed participant may be the focus and foundations of the analysis. For example, in our study, since the focus was set on evaluating the extent to which CT and non-CT utterances changed in the course medical ethics SGL among first-year students, the use of microanalysis fitted perfectly with the purpose of the study. Specifically, our data microanalysis consisted of three main stages. First, each transcribed sentence from the videotaped observations was analyzed using Kamin’s model. The purpose of this basic and fundamental qualitative analysis was to flush out all utterances of the relevant stages of thinking using Kamin’s model, as encapsulated within the transcribed observations. The second stage of the analysis revolved around counting the specific instances of CT] and non-CT thinkingin Kamin’s model per group per session. At this stage, multiple CT and non-CT (, expressed per a single utterance of a single student, were also counted. These utterances, similar to those expressing single CT or non-CT utterances, were also counted per session, per group. The purpose of counting these multiple CT and non-CT per student single expressions was to probe the nature of higher critical thinking utterances versus lower non-critical thinking utterances in each of the group sessions. We considered multiple CT expressions in a single student’s utterance as having 3 and above CT), The thought behind focusing on these multiple CT expressions was that they signify the most illustrious form of critical thinking, since these multiple CT expressions reflect not a single utterance but 3 utterances of critical thinking per a student’s single statement. Initially we also probed for 3 and more multiple non-CT, but since there were no such cases, we opted for 2 non-CT instances per student’s utterance. Finally, in the third stage of the data analysis process, the medians of the first part of the year (before the intervention program) and the second part of the year (after the intervention program) were calculated for all the utterances of CT and non-CT, as well as multiple CT and multiple non-CT. The use of medians rather than averages was chosen to avoid any misrepresentation due to outliers, whether in the direction of CT or non-CT [27].

To ensure the trustworthiness of the qualitative research, an ongoing discussion took place between the two authors about the coding of the various utterances of CT and non-CT. In any cases of disagreement, the particular codlings questioned were discussed, reverting to the relevant portions of the original transcripts of the videotaped observations, until an agreement was reached. a large number of observations were also done on the different groups rather than just a one-time observation, thereby further increasing the trustworthiness of the data analysis overall.

### Ethical considerations

The study was approved by the University Ethics in Research Committee (Approval No 10–2016). At the beginning of the school year, students were given an explanation of the research and the opportunity to decide not to participate or request to withdraw their participation during the course of the research. Students who felt uncomfortable exposing their faces to the camera were allowed to sit in places where the camera would not entirely catch their faces. The videotaped observations were also stored on a password-protected personal computer available only to the first author, and removed from the cameras used for recording the sessions once those videotaped observations were uploaded to that computer.

The transcriptions of the recorded observations also did not include any personally identifiable information. Individual students were identified only through assigned numbers in ascending order (1,2,3, etc.) per group, together with an indication for whether the student was male or female.

## Results

### Demonstration of the first-step (fundamental) microanalysis

Table 1 shows examples for the first-stage data analysis, where the relevant stages of CT and non-CT according to Kamin’s model are assigned to each of the expressions made by the students and facilitators. The first example is taken from the one of the sessions taking place before any intervention was conducted, where the students discussed a case in cultural competence. The second excerpt is from the same session about cultural competence, but this time, the focus is set on multiple CT and non-CT expressions in students’ single utterances. This latter example also includes expressions showing (in our analysis) instances of tolerance and intolerance to ambiguity (AD and AIS, respectively).

**Table 1:**
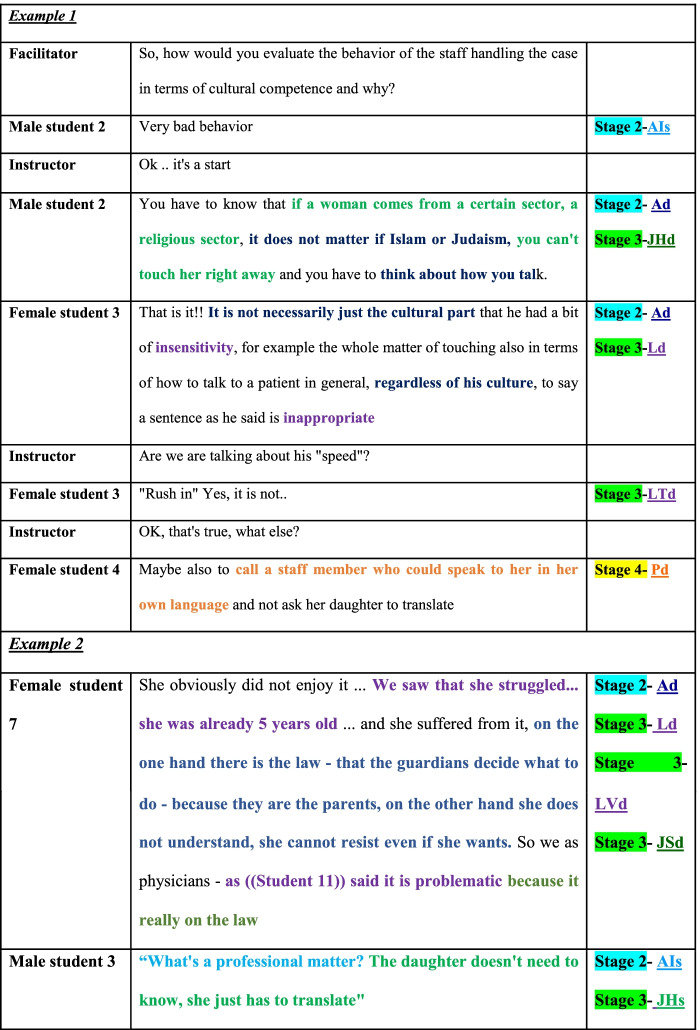
Example for the first-step microanalysis^a^

^a^Ad = discuss ambiguities or facts to clear them up (stage 2 – problem identification [CT]); Ld = linking ideas and facts (stage 3 – problem exploration [CT]); LVd = Interpretation of data (on video) (stage 3 – problem exploration [CT]; JSd = justifying hypotheses or order / action by providing examples or explaining (as part of stage 3 – problem exploration [CT]; AIs = ignoring or exhibiting impatience with ambiguities (stage 2 – problem identification [non-CT]); JHs = Unwilling to explore other possible solutions/explanations for problem (stage 3 – problem exploration [non-CT])

### Overall trends of multiple CT and non-CT expressions – start vs. end of year

**Fig. 1:**
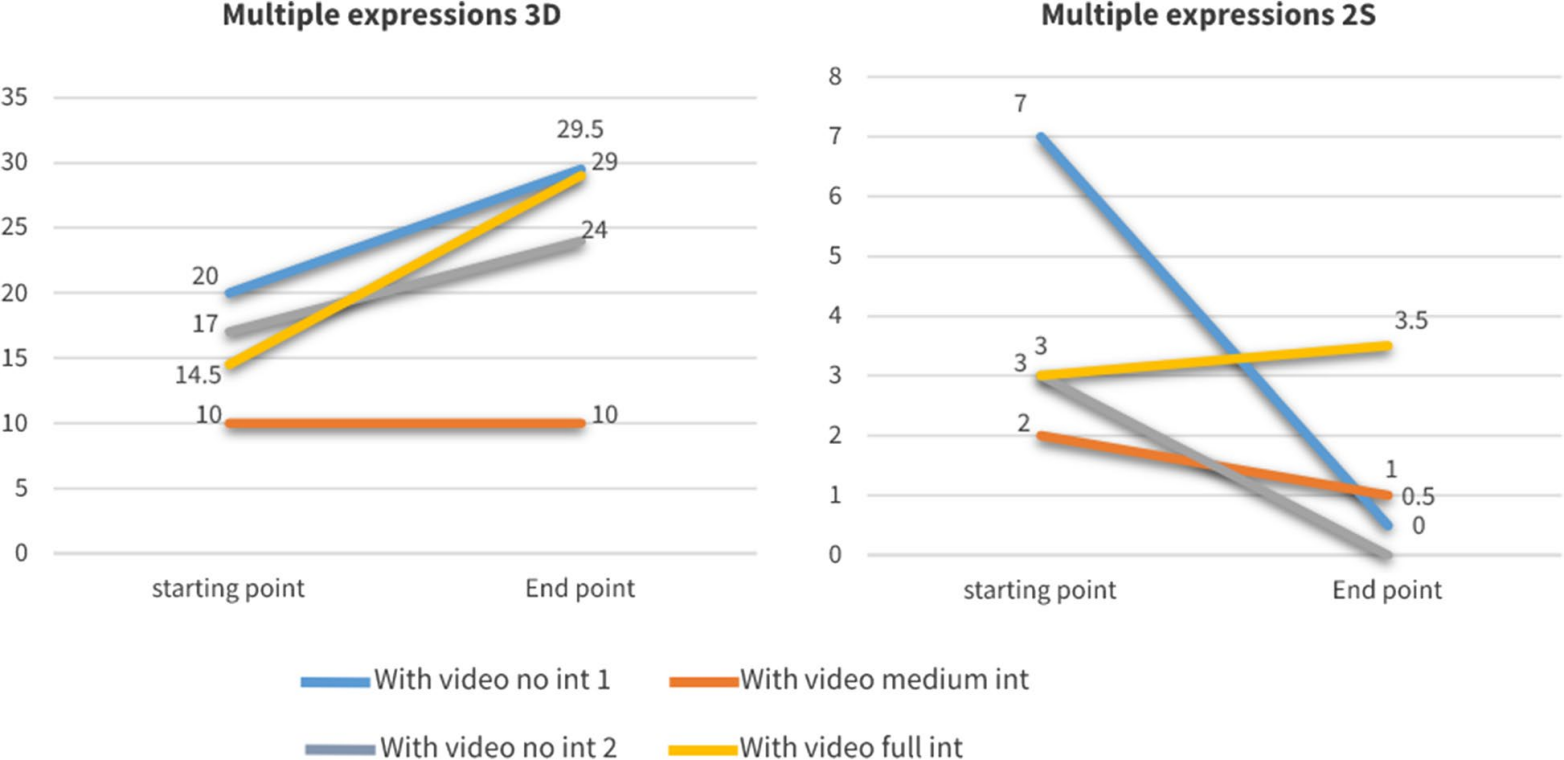
Trends of Multiple Critical Thinking and non-Critical Thinking

The trends of change in multiple CT expressions as well as in multiple non-CT expressions throughout the year were examined in the four groups by comparing the median values of the sessions at the beginning of the year (before the intervention) to the median values in sessions at the end of the year, per group. All these trends are presented, side-by-side in Fig. 1 below.

**Fig. 2:**
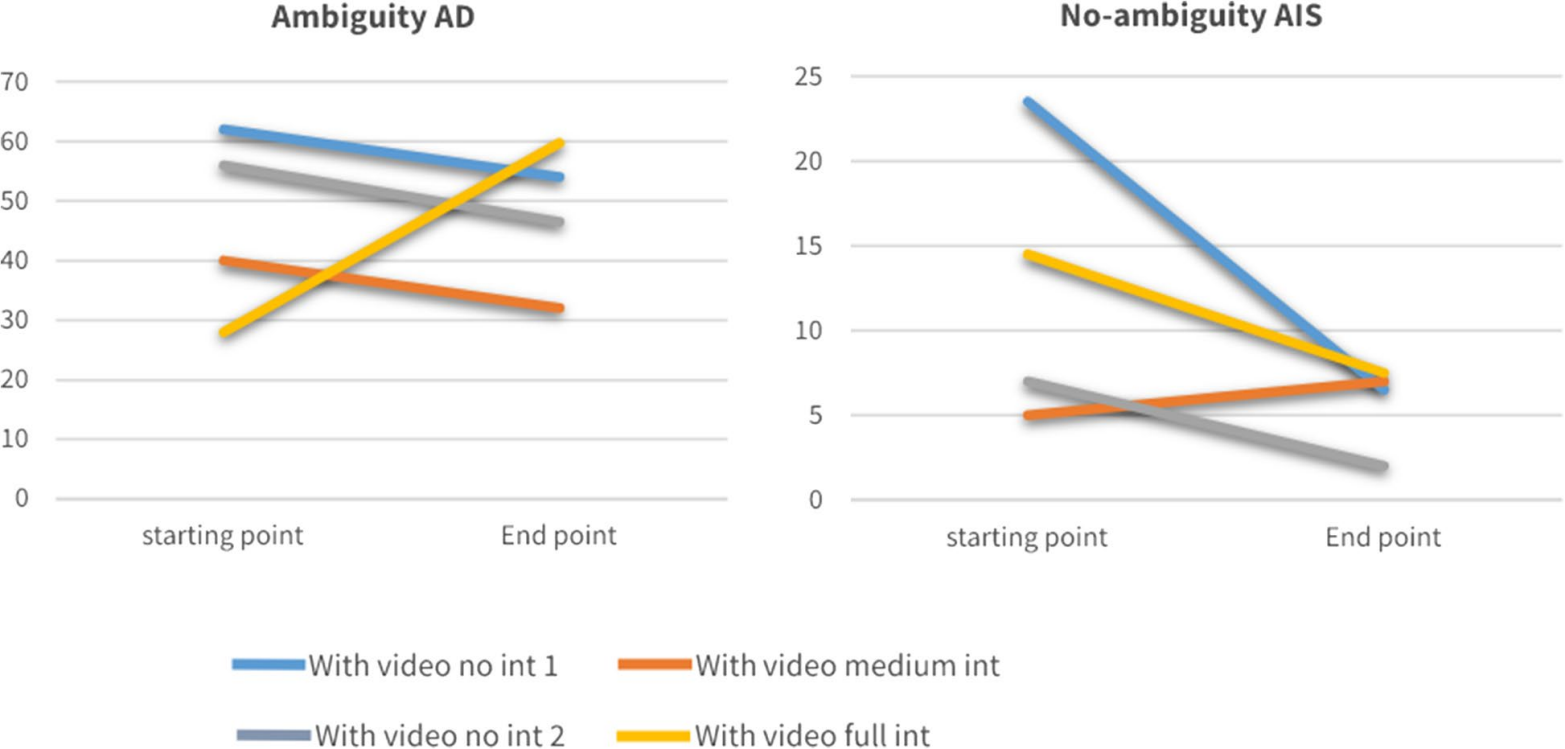
Trends of Tolerance and Intolerance to Ambiguity

Here we can see that most of the groups had a similar baseline at the beginning of the year (median of 15–20 multiple CT expressions per single student’s utterance). Yet, the largest increase in deep (or high) CT is exhibited in the full intervention group (i.e., the exercised debate group), where the median value doubled itself at the end of the year (14.5 vs. 29). However, there is also a slight increase in expressions that manifested non-CT compared with all the other groups that showed actually a decrease in multiple non-CT expressions. Similarly, the trends of the change in tolerance to ambiguity (AD) and intolerance to it (AIS) throughout the year were examined in the four groups. These trends are presented in Fig. 2 below. We can thus see that the debate group is the only one showing an upward trend in the tolerance of ambiguity. However, for expressions that manifested intolerance to ambiguity throughout the year, while there was a decline in the full intervention (debate) group, other groups showed a steeper decline in the use of such expressions.

### Trends in the distribution of participants’ expressing multiple CT

We also wanted to examine the distribution (or dispersion) of the participants who expressed higher levels of CT (multiple CT per a single statement/expression of a student), when comparing the beginning of the year sessions to sessions at the end of the year, per participant (student) in each group. Table 2 presents these findings. To clarify the trends per student, different cell colors were used. ***Green*** - represents an upward trend (beginning ➔end of the year); ***orange*** - represents an unchanged trend; and ***red*** - represented a downward trend.

**Table 2:**
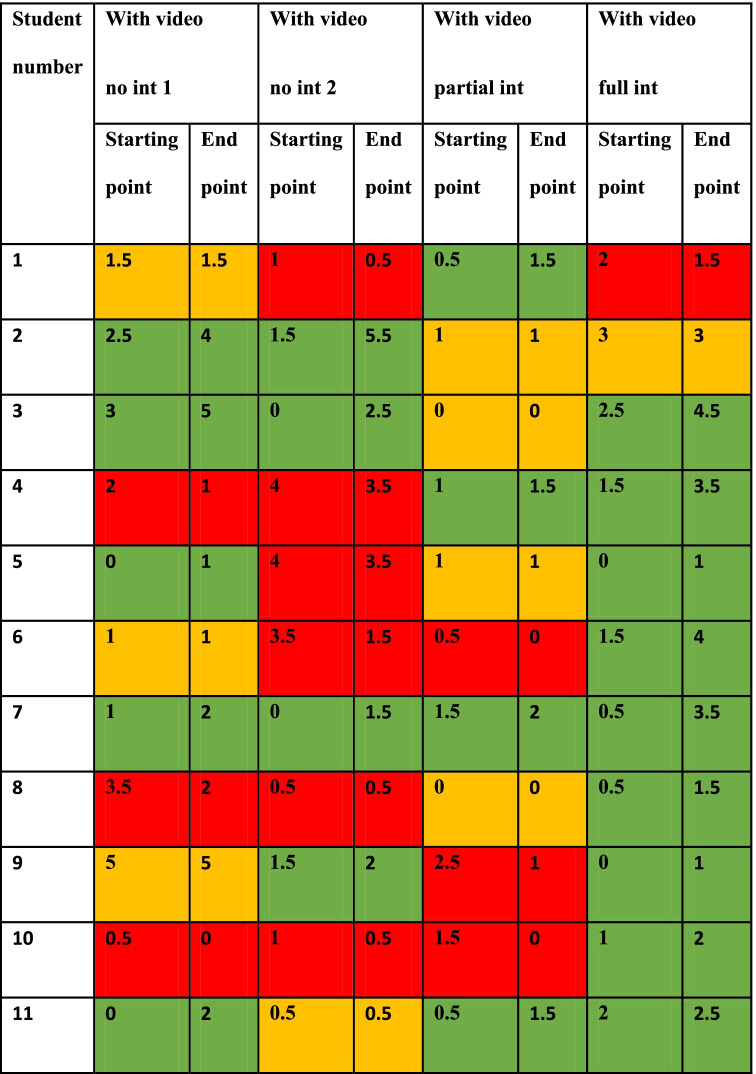
Multiple CT and non-CT per student, per group, at start and end of the year

It was observed that the upward trend (marked in green) stands out in the full intervention (debate) group, compared to the rest of the groups. In the debate team as many as 9 (out of 11) students showed an upward trend in their expression of complex (multiple) CT per student utterance. In contrast, other groups showed merely half the number of participants with upward trends (4–5 students per groups). Hence, the debate group showed a more equal distribution of participation among its members.

## Discussion

Our study demonstrated that debating produced mixed results insofar as CT was concerned. On the one hand, the use of debate improved the students’ CT throughout the year, including a more equal distribution of CT among the students in this group, compared to the other groups. On the other hand, the use of debate also increased the number of superficial or non-critical thinking utterances.

These findings are novel, since previous studies did not examine the possible contribution of the debating tool to the study of medical ethics in a SGL format, and the majority of these studies only measured medical ethics teaching using quantitative indices, mainly self-reported questionnaires by the students [28–30].

Our positive finding on the contribution of debating to the CT of students during the SGL sessions in medical ethics did resonate with and further corroborated existing literature. For instance, it was shown that debating offers key principles in the development of critical thinking, namely, the distinction between opinion and fact and a structured encounter with different opinions [7, 8, 31]. Other studies have showed that debates provide a clear framework for discourse; the participants take a stand and are asked to represent and argue them without fear of personal attack [32, 33]. There is also an active listening behavior aspect since each participant should explain why the other side is wrong and thus needs to listen and relate to their claims [34].

In terms of a more equal distribution of CT among the students in the debating intervention group, the current result makes sense. Hence, the debate method gives instructors clear rules of the game and there is a strict rotation, allowing all opinions to be heard equally and fairly as they are being debated, without breaking the line of thought [15].

Furthermore, previous studies highlighted the possible insignificance that students may attribute to any medical ethics studies [35, 36]. In contrast, the use of debate in the context of educational programs has been associated with a learner’s positive experience and greater participation in the learning process [37, 38]. Against this backdrop, our findings revealed a clear increase in CT during the employment of debating and thus are particularly important, as these findings highlight the positive potential for using debating to increasing actual interest in medical ethics and those classes.

Similarly, the issue of ambiguity is an important prerequisite for a physician’s professionalism, yet studies show that medical students have difficulty accepting ambiguities, as they tend to perceive medical knowledge as being only “clear cut.” [39, 40] “Intolerance toward ambiguity” was first identified more than 70 years ago, and later been described as either a personal trait or a situational judgment where new, complex, or unresolved situations are only seen as “sources of threat.” [41] Therefore, our study’s results, which indicated that the use of debate increased the expression of tolerance toward ambiguity, highlights yet again the potential that debating offers for further enhancing an important key aspect of the medical profession. At the same time, this finding is not surprising, since previous studies on the integration of debate for different subjects found that students did change their opinions during discussions, and that change was based on understanding and acknowledging the existence of multiple interpretations of the same situation [42, 43].

On the other hand, the current study also shows the limits of debating, at least in the context of medical ethics SGL. Hence, the group using debates in their discussions of cases in medical ethics also showed either an increase in multiple non-CT expressions or only a lesser decrease in their intolerance to ambiguity. These limitations of the use of debating in the current examined context also resonated with known critiques about the use of debates as an educational tool. There have been claims made that debates put more emphasis on form than on content. Also, due to the clear framework of the arguments “for” and “against” a certain point of view, learners do not necessarily formulate a self-identity, but rather present arguments only, thereby damaging their present and future internal integrity [44].

That being said, the results of our study do show that debating may still offer an important enhancement or “upgrade” to SGL in medical ethics classes. The increase in CT expressions was more clearly demonstrated in the group that employed debating in the current study.

### Study limitations and future research

This study’s population included medical students who were attending a particular program at a specific Faculty of Medicine. Therefore, undertaking this research in another country, in other medical faculties, or in other programs, might yield other results. In addition, the main research tool of observation is naturally limited in the number of participants that can be included easily. This qualitative research method allows for in-depth understanding of the thinking processes that the participants go through, but it cannot be generalized because of the usual small number of participants and the non-statistical nature of this research process.

Future research might benefit from being conducted in more venues around the world and examining additional groups that will allow for gender-relevant comparisons, cultural backgrounds, ages, etc. In addition, interviews with students and facilitators might shed additional light on other or different dimensions and characteristics of the individual or group processes that our videotaped observations did uncover in this study.

## Conclusion

The study reported on in this paper sought to examine the possible contributions of debating to CT in the context of SGL of medical ethics. Using an observation-based qualitative research method that utilized a 3-step microanalysis, the study revealed that CT substantially increased when debating was used. However, these findings do have their limitations, since it was also observed that when debating was used, there was also an increase in non-CT measurements. Hence, the employment of debating does not guarantee the use of only CT. Still, the results of this study do show that debating may indeed offer an important enhancement or “upgrade” to SGL in medical ethics classes.

## Data Availability

The datasets generated or analyzed during this study are not publically available since they use foreign language. However, the datasets are available from the corresponding author on reasonable request.

## Abbreviations

SGL: Small-Group Learning
CT: Critical Thinking

## Footnotes

1 Please keep in mind, that the larger research project included a quantitative portion based on questionnaires, in which the attitudes and perceptions of SGL of medical ethics were explored, and since we thought that the filming/videotaping of sessions might be an intervening factor, 3 groups of SGLs (approx. Half of the students) were purposely not filmed/videotaped.
